# Rapid mass production of two-dimensional metal oxides and hydroxides via the molten salts method

**DOI:** 10.1038/ncomms15630

**Published:** 2017-05-30

**Authors:** Zhimi Hu, Xu Xiao, Huanyu Jin, Tianqi Li, Ming Chen, Zhun Liang, Zhengfeng Guo, Jia Li, Jun Wan, Liang Huang, Yanrong Zhang, Guang Feng, Jun Zhou

**Affiliations:** 1 Wuhan National Laboratory for Optoelectronics, Huazhong University of Science and Technology, Wuhan, Hubei 430074, China; 2 State Key Laboratory of Coal Combustion, School of Energy and Power Engineering, Huazhong University of Science and Technology, Wuhan, Hubei 430074, China; 3 Environmental Science Research Institute, Huazhong University of Science and Technology, Wuhan, Hubei 430074, China

## Abstract

Because of their exotic electronic properties and abundant active sites, two-dimensional (2D) materials have potential in various fields. Pursuing a general synthesis methodology of 2D materials and advancing it from the laboratory to industry is of great importance. This type of method should be low cost, rapid and highly efficient. Here, we report the high-yield synthesis of 2D metal oxides and hydroxides via a molten salts method. We obtained a high-yield of 2D ion-intercalated metal oxides and hydroxides, such as cation-intercalated manganese oxides (Na_0.55_Mn_2_O_4_·1.5H_2_O and K_0.27_MnO_2_·0.54H_2_O), cation-intercalated tungsten oxides (Li_2_WO_4_ and Na_2_W_4_O_13_), and anion-intercalated metal hydroxides (Zn_5_(OH)_8_(NO_3_)_2_·2H_2_O and Cu_2_(OH)_3_NO_3_), with a large lateral size and nanometre thickness in a short time. Using 2D Na_2_W_4_O_13_ as an electrode, a high performance electrochemical supercapacitor is achieved. We anticipate that our method will enable new path to the high-yield synthesis of 2D materials for applications in energy-related fields and beyond.

## Abstract (web summary)

2D materials with exotic electronic properties are increasingly important for the development of low-dimensional electronic devices. Here, Hu *et al*. have developed a fast and efficient method to synthesize 2D metal oxides and hydroxides, further enabling 2D electronics.

Because of their thickness-dependent properties, two-dimensional (2D) atomic crystals are of great interest in various applications ranging from superconductors and field effect transistors to electrocatalysis and energy storage^1^,^2^,^3^,^4^,^5^,^6^,^7^,^8^. Current strategies for the synthesis of 2D materials primarily include exfoliation, chemical vapour deposition (CVD) and chemical synthesis^9^,^10^,^11^,^12^,^13^,^14^,^15^,^16^,^17^,^18^. Exfoliation is applied on layered compounds, such as graphene and MoS_2_, in which each layer is bonded by van der Waals forces^9^,^19^,^20^,^21^,^22^. Recently, Coleman *et al*. reported a shear exfoliation method for the scalable production of high-quality 2D materials^9^,^21^,^22^. Wu *et al*.^23^ also accomplished the mass production of oxide nanosheets by a rapid thermal annealing treatment of the hydrous-chloride compounds. However, the further centrifugation and sedimentation necessary to separate the unexfoliated bulk complicate the process, increase the cost and decrease the yield. The CVD method can obtain high-quality and large lateral size 2D materials, although the low yield of the CVD method confines its applications mainly to physics and optics^10^,^11^. High-yield chemical synthesis is widely studied in energy-related fields. Generally, a surfactant such as polyvinyl pyrrolidone (PVP) is used to guide the growth of 2D materials, however, the removal of these surfactants is time consuming and sometimes not completely successful. In addition, it should be noted that chemical synthesis usually requires a long amount of time, even several days, which is not favourable for practical applications^12^. Therefore, it is of crucial significance to develop a high-yield, efficient, fast and low-cost method to synthesize 2D materials.

Notably, ions always play a key role in the synthesis of 2D materials^24^,^25^,^26^,^27^,^28^. For example, Li^+^ ions are pre-intercalated into the interlayers of MoS_2_, increasing the interlayer distance and facilitating the exfoliation of the layered materials^24^,^28^. By contrast, for ion-intercalated 2D materials such as *δ*-MnO_2_ (birnessite), desolvated ions can serve as the template to induce the growth in the 2D plane^25^,^26^,^27^. Interestingly, these pre-intercalated desolvated ions are favourable for energy storage and show unique properties in optics^29^. However, it should be noted that when the synthesis process occurs in solution, desolvation is a necessary step because ions are in the solvated state in solution. In this context, according to the Arrhenius equation (ref. 30), the energy consumption for desolvation increases the overall activation energy, thus limiting the reaction rate. In addition, incompletely desolvated ions cause lattice distortion, which decreases the quality of 2D materials. Accordingly, we can reasonably assume that if naked ions can be directly involved in a synthesis process, the reaction limitation due to the desolvation of hydrated ions will be alleviated, which will greatly accelerate the reaction rate and enhance the quality of the generated 2D materials.

Herein, we report a general and rapid molten salts method (MSM) that can synthesize various ion-intercalated 2D metal oxides and hydroxides, such as cation-intercalated manganese oxides (Na_0.55_Mn_2_O_4_·1.5H_2_O and K_0.27_MnO_2_·0.54H_2_O), cation-intercalated tungsten oxides (Li_2_WO_4_ and Na_2_W_4_O_13_), and anion-intercalated metal hydroxides (Zn_5_(OH)_8_(NO_3_)_2_·2H_2_O and Cu_2_(OH)_3_NO_3_). The MSM is widely studied for the synthesis of nanomaterials, such as graphene and perovskite, spinel and monoclinic oxides^31^,^32^,^33^,^34^. The key feature of our method is the direct use of naked ionized ions without hydration in the molten state salt to quickly induce the growth of 2D metal oxides and hydroxides. In our technique, by adding precursors into the low-cost molten salts for only 1 min, we could obtain high-yield 2D materials simply by washing the salts without centrifugation or sedimentation, and the productivity of Na_0.55_Mn_2_O_4_·1.5H_2_O reaches up to 62% (the method of calculating the productivity is shown in Supplementary Note 1).

## Results

### Synthesis of 2D metal oxides and hydroxides

**Figure 1:**
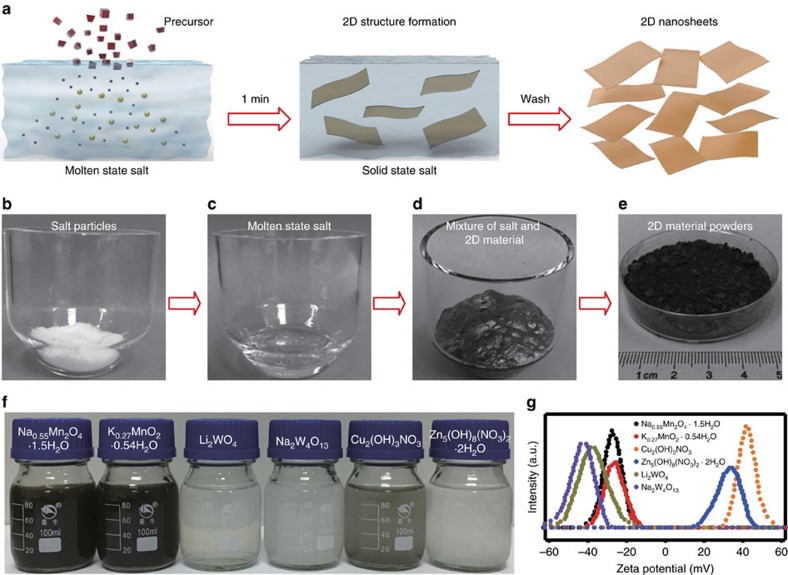
Synthesis process and 2D materials dispersion. (**a**) Schematic of the MSM synthesis of 2D oxides and hydroxides. First, salts were heated to the molten state in a muffle furnace, and then precursors were added into them. Second, after reacting for 1 min, we removed the samples from the furnace. Finally, after washing the salts with DI water, 2D oxides and hydroxides nanosheets were obtained. (**b**) Image of NaNO_3_ particles in a 50 ml quartz crucible. (**c**) Image of molten salt NaNO_3_ at 350 °C. (**d**) Image of a mixture of the salt and 2D Na_0.55_Mn_2_O_4_·1.5H_2_O after reacting for 1 min. (**e**) Image of 2D Na_0.55_Mn_2_O_4_·1.5H_2_O powder after washing with DI water. (**f**) Image of different 2D oxides and hydroxides dispersed in DI water at a concentration of 0.2 mg ml^−1^. (**g**) Zeta potentials of various 2D oxides and hydroxides in DI water.

Salts are common ionic crystals that can ionize in the molten state. In our experiments, we typically started by heating the nitrate to the molten state in a muffle furnace and adding the precursor into it (Fig. 1a–c). Here, the ionized species from the molten salts rapidly react with the precursor to form the 2D structured materials, at which point an apparent expansion was observed in the mixture (this mixture contains 2D materials, recrystallized salts and by-products) (Fig. 1d, Supplementary Fig. 1 and Supplementary Movie 1). After cooling down and washing the salts via filtration, we could directly obtain the 2D materials (Fig. 1e) or disperse them in deionized water (DI water) for further applications. Figure 1f shows digital image of various 2D metal oxides and hydroxides dispersions. All of these 2D materials dispersed uniformly in DI water at a concentration of 0.2 mg ml^−1^. As shown in Fig. 1g, we tested the zeta potential of these dispersions. Cation-intercalated 2D metal oxides show negative zeta potentials, while anion-intercalated metal hydroxides have positive zeta potentials. The highest value of about −42 mV is achieved in Na_2_W_4_O_13_, and the smallest value is around −22 mV in K_0.27_MnO_2_·0.54H_2_O.

**Figure 2:**
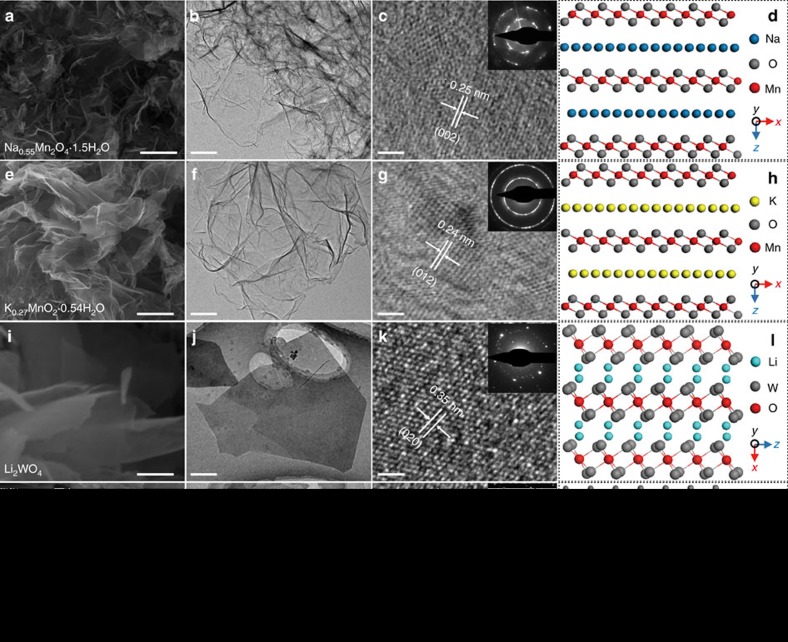
Characterization of cation intercalated 2D oxides. Characterization of Na_0.55_Mn_2_O_4_·1.5H_2_O. (**a**) SEM image; (**b**) low-resolution TEM image; (**c**) high-resolution TEM image; (**d**) atomic structure. Characterization of K_0.27_MnO_2_·0.54H_2_O. (**e**) SEM image; (**f**) low-resolution TEM image; (**g**) high-resolution TEM image; (**h**) atomic structure. Characterization of Li_2_WO_4_. (**i**) SEM image; (**j**) low-resolution TEM image; (**k**) high-resolution TEM image; (**l**) atomic structure. (**d**) Characterization of Na_2_W_4_O_13_. (**m**) SEM image; (**n**) low-resolution TEM image; (**o**) high-resolution TEM image; (**p**) atomic structure. Scale bar for **a**,**e**,**i**,**m** is 1 μm, **b**,**f**,**j**,**n** is 200 nm and **c**,**g**,**k**,**o** is 2 nm.

To explore the morphology and structure of these 2D materials, we conducted scanning electron microscopy (SEM) and transmission electron microscopy (TEM). As shown in Fig. 2a,e,i,m, 2D materials are clearly seen in the representative SEM images. All of them show 2D structures, but with different morphologies, which can also be seen in TEM images (Fig. 2b,f,j,n). Na_0.55_Mn_2_O_4_·1.5H_2_O and K_0.27_MnO_2_·0.54H_2_O are very flexible and have apparent wrinkles, while Li_2_WO_4_ and Na_2_W_4_O_13_ seem to be stiff. However, they all display high transparency and have thicknesses less than 5 nm (Supplementary Figs 2 and 3). In addition, high-resolution TEM (HRTEM) and selected area electron diffraction (SAED) images demonstrate the crystallinity of these 2D materials (Fig. 2c,g,k,o). Na_0.55_Mn_2_O_4_·1.5H_2_O and K_0.27_MnO_2_·0.54H_2_O are polycrystalline, while Li_2_WO_4_ and Na_2_W_4_O_13_ are single crystalline. Furthermore, we used energy dispersive X-ray spectroscopy (EDS, Supplementary Fig. 4), X-ray diffraction (XRD, Supplementary Fig. 5) and X-ray photoelectron spectroscopy (XPS, Supplementary Figs 6–8) to confirm the elemental composition and atomic structure. All of these 2D metal oxides show similar layered structures with cations intercalated into the interlayer (Fig. 2d,h,l,p, Supplementary Fig. 9). Na_0.55_Mn_2_O_4_·1.5H_2_O has a monoclinic structure (*a*=5.175 Å, *b*=2.849 Å and *c*=7.338 Å) with Na^+^ and crystal water filling the interlayer. The thickness of the monolayer is 7.3 Å, and the sample we synthesized has ∼2 layers (∼1.4 nm, Supplementary Fig. 2). K_0.27_MnO_2_·0.54H_2_O possesses a rhombohedral structure with *a*=2.849 Å, *b*=2.849 Å and *c*=21.536 Å. The sample thickness of about 1.4 nm (Supplementary Fig. 2) is ∼2 layers compared with the thickness of the monolayer (7.18 Å). Li_2_WO_4_ is a rhombohedral layered material with Li^+^ intercalated in the interlayer (*a*=14.361 Å, *b*=14.361 Å and *c*=9.603 Å) and a monolayer thickness of 4.80 Å. According to measurements using atomic force microscopy (AFM, Supplementary Fig. 3), the synthesized Li_2_WO_4_ is ∼9–10 layers in thickness. Na_2_W_4_O_13_, however, has a different atomic structure than Li_2_WO_4_. It has an anorthic structure (*a*=8.381 Å, *b*=8.162 Å and *c*=3.87 Å) with a monolayer thickness of 4.19 Å. We tested the thickness of Na_2_W_4_O_13_ and found that it was approximately 3.79 nm, which is ∼9 layers (Supplementary Fig. 3).

**Figure 3:**
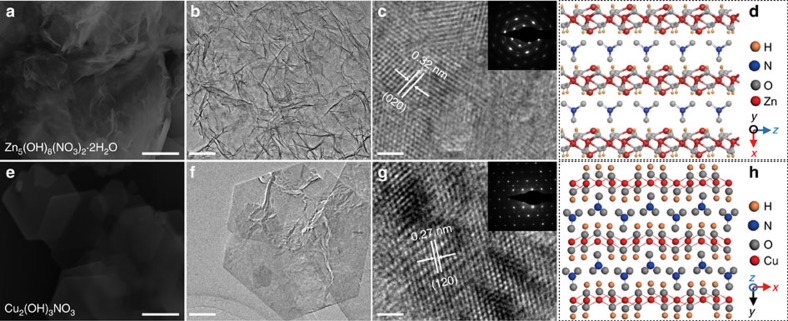
Characterization of anion intercalated 2D hydroxides. Characterization of Zn_5_(OH)_8_(NO_3_)_2_·2H_2_O. (**a**) SEM image; (**b**) low-resolution TEM image; (**c**) high-resolution TEM image; (**d**) atomic structure. Characterization of Cu_2_(OH)_3_NO_3_. (**e**) SEM image; (**f**) low-resolution TEM image; (**g**) high-resolution TEM image; (**h**) atomic structure. Scale bar for **a**,**e** is 1 μm, **b**,**f** is 200 nm and **c**,**g** is 2 nm.

Analogous to cations in cation-intercalated metal oxides, anions such as NO_3_^−^ can also be used as the template to induce the synthesis of anion-intercalated metal hydroxides. Here, we used the MSM to successfully synthesize 2D NO_3_^−^-intercalated Zn hydroxide and Cu hydroxide. As shown in SEM and TEM images (Fig. 3a,b,e,f), they both exhibited typical 2D structures. In addition, HRTEM and SAED (Fig. 3c,g) demonstrated the high crystallinity of the as-synthesized anion-intercalated metal hydroxides. The atomic structures were confirmed by XRD, as shown in Fig. 3d,h and Supplementary Figs 10 and 11. Zn_5_(OH)_8_(NO_3_)_2_·2H_2_O has a monoclinic structure with *a*=19.48 Å, *b*=6.238 Å and *c*=5.517 Å. NO_3_^−^ and crystal water occupied the interlayer with a monolayer thickness of 9.74 Å. Hence, the as-synthesized Zn_5_(OH)_8_(NO_3_)_2_·2H_2_O is ∼2 layers thick (about 2 nm) (Supplementary Fig. 12). Cu_2_(OH)_3_NO_3_ is also monoclinic with *a*=5.605 Å, *b*=6.087 Å and *c*=6.929 Å. The monolayer thickness is 6.93 Å, which suggests that our synthesized 2D Cu_2_(OH)_3_NO_3_ has approximately five layers (Supplementary Fig. 13).

### Growth mechanism

**Figure 4:**
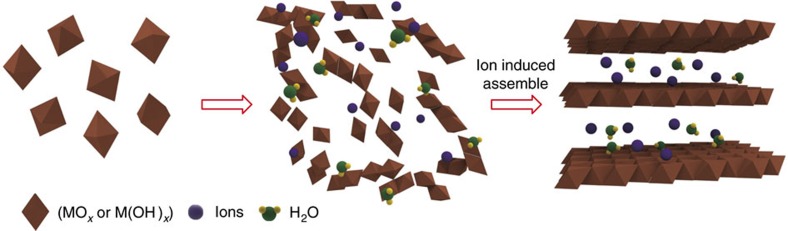
Proposed mechanism of the molten salts synthesis of 2D ion-intercalated metal oxides and hydroxides. MO_*x*_ or M(OH)_*x*_ (M represents metal) molecules were formed when the metal ions from the precursor reacted with nitrate or H_2_O. During the self-assembly of these MO_*x*_ or M(OH)_*x*_ molecules, ions from the molten salts rapidly arranged in a 2D plane and guided the growth of the 2D structure.

We then examined the growth mechanism of our MSM for 2D ion-intercalated metal oxides and hydroxides. As we previously mentioned, all of these layer-like 2D metal oxides and hydroxides have similar atomic structures in which ions intercalate in the interlayer. Hence, we proposed that here, the ions play a key role in the synthesis process^25^,^26^,^35^,^36^. The formation of 2D layered metal oxides and hydroxides consists of two steps (Fig. 4). The first step is the nucleation of [MO_*x*_]_octahedron_ or [M(OH)_*x*_]_polyhedron_ seeds (M represents metal ion, *x* is the number of oxygen atoms), and the second step is the growth of crystal based on the assembly of these [MO_*x*_]_octahedron_ or [M(OH)_*x*_]_polyhedron_ seeds influenced by the ions from the molten salts (Fig. 4). For the synthesis of 2D Na_0.55_Mn_2_O_4_·1.5H_2_O and K_0.27_MnO_2_·0.54H_2_O, [MnO_6_]_octahedron_ will be formed via the following redox reaction (equation (1)) in the first step (*y* is the number of NO_3_^−^ ions):


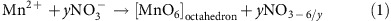


Then, these [MnO_6_]_octahedron_ seeds assemble into a 2D plane with the cations and H_2_O molecules (H_2_O originates from the atmosphere; see details in Supplementary Note 2 and in Supplementary Fig. 14) intercalated into the interlayer to balance the charge and stabilize the layered structure^25^,^26^ and formed the 2D ion-intercalated metal oxides (Supplementary Fig. 15) according to equations (2) and (3):


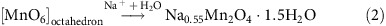



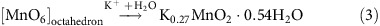


For the formation of 2D Zn_5_(OH)_8_(NO_3_)_2_·2H_2_O, Zn^2+^ ions first react with H_2_O to form [Zn(OH)_6_]_octahedron_ and [Zn(OH)_4_]_tetrahedron_ via a hydrolysis reaction (equation (4), *m* is the number of H_2_O). Then, the [Zn(OH)_6_]_octahedron_ and [Zn(OH)_4_]_tetrahedron_ seeds assemble into a 2D plane with the anions and H_2_O molecules intercalated into the interlayer, which balances the charge and stabilizes the layered structure (equation (5) and Supplementary Fig. 16)^37^,^38^,^39^. The formation of 2D Cu_2_(OH)_3_NO_3_ occurs via a similar process, as shown in equations (6) and (7) and Supplementary Fig. 17.


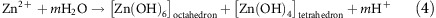



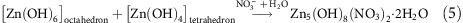



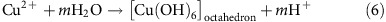



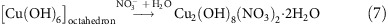


Now, we discuss why only 2D morphology, not one-dimensional (1D) or three-dimensional (3D) morphologies, was obtained in this study. Here, we further explain the example of birnessite (K_0.27_MnO_2_·0.54H_2_O) in detail. MnO_2_ exists as various types of crystal structures, such as *α*, *β*, *γ* and *δ* phases, all of which can be considered as kinetically stable phases. It is agreed that birnessite-type MnO_2_ is the kinetically favoured product and is always formed first, while tunnelled-structure MnO_2_ species, such as *α* phase and *β* phase, are the thermodynamically favoured products and can be formed from *δ*-MnO_2_ (ref. 40). After the reaction time was extended to 10 min, the crystal structure and 2D morphology features of *δ*-MnO_2_ remain unchanged (Supplementary Fig. 18). Hence, 2D ion-intercalated MnO_2_ is stable under the reaction conditions in this work.

Compared to previously reported hydrothermal syntheses of birnessite^26^,^35^, there are two obvious differences in our method: relatively higher temperature (∼350 °C) and ionized species (without hydration). Generally, the reaction temperature and the ion state can directly influence the morphology of the products. Thus, we discuss the influence of these two factors on the morphology of the 2D materials in detail. First, we determined the relationship between reaction temperature (K) and reaction rate constant (*k*_*a*_ and *k*_*b*_, which represent the reaction rate constant in growth direction [001] (thickness) and [100] (lateral size), respectively). In a typical 2D structured material, the reaction rate constant in different growth directions determines the thickness and lateral size.

According to the Arrhenius equation^30^, we can obtain the relationship of *k*_*a*_ and *k*_*b*_ (see details in Supplementary Note 3):


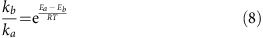


where *E*_*a*_ and *E*_*b*_ are the activation energy in different growth directions (J mol^−1^), *R* is the molar gas constant (8.314 J mol^−1^ K^−1^) and *T* is the reaction temperature (K). According to equation (8), if we only increase the reaction temperature, the value of *k*_*b*_/*k*_*a*_ will decrease (see details in Supplementary Note 4), signifying that the thickness of 2D *δ*-MnO_2_ increases faster than the lateral size increases. Thus, a large lateral size with a subnanometer thickness cannot be obtained, which is not consistent with our experimental results, as shown in Figs 2 and 3. Hence, we could exclude the temperature as the main factor.

Second, we compared the activation energy along the [100] direction in our method (*E*_*b*′_) to that in ref. 41 (*E*_*b*_) (see details in Supplementary Note 5): *E*_*b*_−*E*_*b*′_=23.9–32.0 kJ mol^−1^. As the total hydration energy of K^+^ is −312.2 kJ mol^−1^ (that is, a fully hydrated K^+^ ion requires 312.2 kJ mol^−1^ of energy to form a naked ion; see details in Supplementary Note 6), the K^+^ number is 0.27 in our work, thus, the dehydration energy is ∼84.3 kJ mol^−1^ for the sample of K_0.27_MnO_2_·0.54H_2_O. Considering that the dehydration process does not go to completion in the hydrothermal system, this value has the same order of magnitude as the value of (*E*_*b*_−*E*_*b*′_), for example, the actual dehydration energy would be lower than 84.3 kJ mol^−1^. Accordingly, it is reasonable that the ionized species in our method affect the size of the 2D materials by accelerating the reaction rate (do not need the dehydration process).

In addition, we also noted that the type of ion is important for the synthesis of 2D birnessite. Other types of ion-intercalated 2D manganese oxides can be obtained by choosing the appropriate molten salts, that is, those beyond NaNO_3_ and KNO_3_. By using MnSO_4_ as the precursor, 2D Cs_4_Mn_14_O_27_·*x*H_2_O and 2D Ba_2_Mn_14_O_27_·*x*H_2_O can be synthesized from the molten salts of CsNO_3_ and Ba(NO_3_)_2_ (Supplementary Fig. 19), respectively. However, if we use LiNO_3_ as the molten salt, *β-*MnO_2_ nanowires instead of 2D nanosheets will be obtained (Supplementary Fig. 20). This is maybe because the radius size of the desolvated Li^+^ is too small to support the interlayer of birnessite (about 0.7 nm), which demonstrates that the template effect of ions is of great importance to the synthesis of 2D materials^26^,^27^.

### Applications of 2D Na_2_W_4_O_13_

Because of the large interlayer spacing of these 2D materials, the ions could easily diffuse in or out, which is beneficial for energy storage application. Moreover, 2D morphologies have a quite large specific surface area (Supplementary Fig. 21, Supplementary Table 1) that can provide more active sites. Here, we investigate the electrochemical properties of 2D Na_2_W_4_O_13_ as an example (Supplementary Figs. 22,23 and Supplementary Note 7). First, we used a three-electrode configuration in Swagelok cell to test electrochemical performance. An electrode was fabricated by vacuum-filtrating the 2D Na_2_W_4_O_13_ dispersion onto a Celgard film. The thickness of the film was ∼1 μm (inset of Supplementary Fig. 22b). The Na_2_W_4_O_13_ electrode has excellent wetting behaviour (Supplementary Fig. 24), which provides favourable contact with the electrolyte (0.5 mol l^−1^ Na_2_SO_4_ solution). The restacked 2D Na_2_W_4_O_13_ was tested as a positive electrode and revealed typical pseudocapacitive behaviour, as shown in cyclic voltammetry (CV) curves at sweep rates ranging from 5 to 100 mV s^−1^ (Supplementary Fig. 22a). The largest volumetric capacitance is ∼310 F cm^−3^ at 5 mV s^−1^, whereas 200 F cm^−3^ is still obtained at 100 mV s^−1^, thus showing a good rate capability. Moreover, the restacked 2D Na_2_W_4_O_13_ electrode demonstrated good stability since after 10,000 cycles at 10 mV s^−1^, the capacitance still remained at 93% of its initial value (Supplementary Fig. 22c).

**Figure 5:**
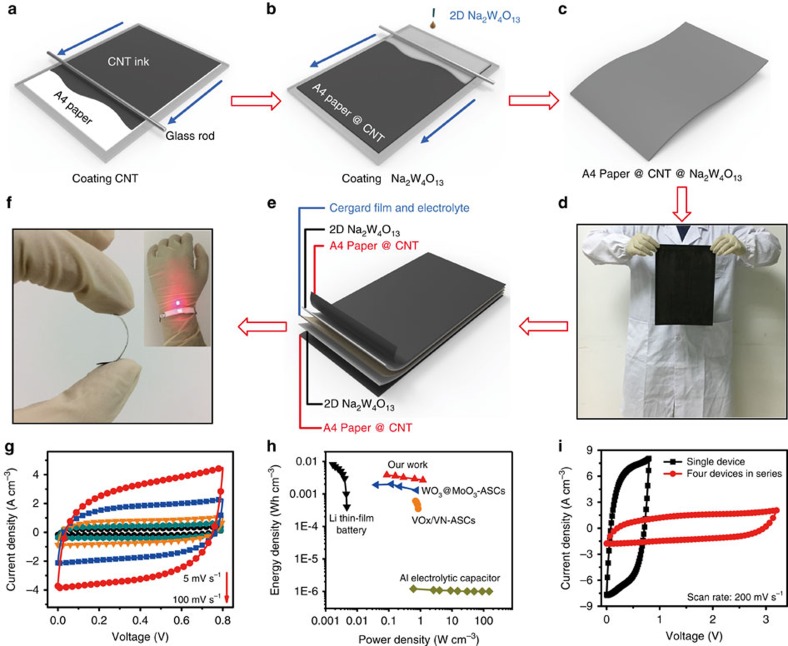
Fabrication and electrochemical performance of solid-state supercapacitors based on 2D Na_2_W_4_O_13_. (**a**) Coating CNTs. We dropped CNT ink on A4 paper and used the Mayer rod method (glass rod) to coat the CNTs on the A4 paper uniformly. (**b**,**c**) Coating Na_2_W_4_O_13_. We dropped the Na_2_W_4_O_13_ suspension on the A4 paper and used a glass rod to coat Na_2_W_4_O_13_ on the A4 paper@CNT. (**d**) Image of the A4 paper@CNT@Na_2_W_4_O_13_. (**e**) Schematic of the A4 paper@CNT@Na_2_W_4_O_13_-based solid-state supercapacitor, showing the different functional layers. (**f**) Image of the solid-state supercapacitor in the bending state. The inset is the four supercapacitors connected in series that can power a red commercial LED. (**g**) CV curves at different sweep rates of the solid-state supercapacitor. (**h**) Ragone plot of the solid-state supercapacitor. (**i**) CV curves at a sweep rate of 200 mV s^−1^ of the single supercapacitor and four supercapacitors connected in series.

In addition, the 2D Na_2_W_4_O_13_ dispersion is uniform enough that the colloidal state of the dispersion was demonstrated by an obvious Tyndall effect (Supplementary Fig. 25a). This dispersion is very suitable for printable electronics. Accordingly, we fabricated a flexible paper-based electrode by coating the 2D Na_2_W_4_O_13_ dispersion on A4 paper. First, we used the Mayer rod method^42^ to coat a uniform CNT layer onto the A4 paper, as a current collector (Fig. 5a). Then, the 2D Na_2_W_4_O_13_ dispersion was coated onto the A4 paper@CNT by the same process (Fig. 5b). Finally, we obtained the flexible A4 paper@CNT@Na_2_W_4_O_13_ electrode, as shown in Fig. 5c,d. The resistivity of the electrode was also measured to be 52.88 Ω, as shown in Supplementary Fig. 25b. The structure schematic of the solid-state SC is illustrated in Fig. 5e. Two A4 paper@CNT@Na_2_W_4_O_13_ electrodes were sandwiched by a Celgard film and a H_3_PO_4_/Polyvinyl Alcohol (PVA) solid-state electrolyte, which resulted in a flexible solid-state supercapacitor (Fig. 5f). According to the CV curves at different sweep rates, this solid-state supercapacitor worked at a stable voltage window from 0 to 0.8 V with typical capacitive behaviour. Even at a high sweep rate of 100 mV s^−1^, only a little distortion could be seen in the CV curve, implying a high ion and electron transport rate (Fig. 5g). Similar to the results from the three-electrode configuration, the solid-state supercapacitor also shows an excellent rate capability as 69% of the initial volumetric capacitance was maintained from 5 to 100 mV s^−1^ (Supplementary Fig. 26). In addition, comparing the CV curves of the A4 paper@CNT electrodes to those of the A4 paper@CNT@Na_2_W_4_O_13_ electrodes under the same sweep rates (Supplementary Fig. 27) suggested very little capacitance contribution from the CNT substrate. The power and energy density are summarized in a Ragone plot, as shown in Fig. 5h. The highest energy density is 3.83 mWh cm^−3^ (1.33 Wh kg^−1^) with a power density of 1.2 W cm^−3^ (598.4 W kg^−1^) (Supplementary Fig. 28 and Supplementary Note 8), comparable to those of a 4 V/500 μAh Li thin-film battery and WO_3_@MoO_3_-ASCs and much higher than those of VO_*x*_/VN-ASCs and an Al electrolytic capacitor^43^,^44^,^45^. Furthermore, we connected four solid-state supercapacitors in series to expand the working voltage window to 3.2 V, as shown in Fig. 5i. After being fully charged, the energy pack could power a commercial red light-emitting-diode (LED, inset of Fig. 5f), revealing the potential applications of the supercapacitors in wearable and portable electronics.

Ion-intercalated tungsten oxide is also a good candidate for ion adsorption. Therefore, we tested the ion adsorption ability of 2D Na_2_W_4_O_13_ in solution. As shown in Supplementary Fig. 29, 2D Na_2_W_4_O_13_ demonstrated an obvious adsorption of Mg^2+^, Cd^2+^, Ca^2+^ and Pb^2+^.

## Discussion

To promote the translation of 2D materials from the laboratory to industry, pursuing a high-efficiency and low-cost synthesis is of great importance. Our MSM was inspired by the low cost of the salts we used, as well as the fact that ionized species naturally exist in the molten state salts and can rapidly induce the growth of 2D materials. Although a certain temperature of 300–400 °C is required, the whole reaction time is just 1 min due to fast ion transport, which decreases the cost. Importantly, we have calculated the productivity of Na_0.55_Mn_2_O_4_·1.5H_2_O to be as high as 62%. In addition, the final mixtures consist of the targeted products (2D materials), unreacted precursors and other salts. The 2D materials can be easily separated from the other species by rinsing the mixtures with DI water. Even without centrifugation or sedimentation, we do not observe particles or nanowires in the final products (SEM images in Figs 2 and 3). This could dramatically simplify the process which is preferable for commercialization. Although we only reported eight 2D ion-intercalated metal oxides and hydroxides, the versatility of this molten salt synthesis process gives us confidence that reasonably tuning the precursors and molten salts will allow various 2D oxides and hydroxides to be synthesized and the scope of the accessible oxides and hydroxides to expand to other 2D materials with attractive properties.

In conclusion, we have reported a rapid mass production molten salts method that can rapidly provide 2D ion-intercalated metal oxides and hydroxides. The ionized species in the molten salts were directly involved in the reaction with the precursors, guiding the growth of the 2D structures within only 1 min. The as-synthesized 2D metal oxides and hydroxides have a large lateral size with an atomic scale thickness. When used as supercapacitor electrodes, these oxides and hydroxides can be fabricated into a flexible solid-state supercapacitor based on A4 paper@CNT@Na_2_W_4_O_13_ electrodes. These show good electrochemical performance with an excellent rate capability, demonstrating the potential applications of these 2D ion-intercalated metal oxides and hydroxides in energy storage and beyond.

## Methods

### Synthesis of 2D Na_0.55_Mn_2_O_4_·1.5H_2_O and K_0.27_MnO_2_·0.54H_2_O

The 2D Na_0.55_Mn_2_O_4_·1.5H_2_O and K_0.27_MnO_2_·0.54H_2_O were synthesized by the MSM. Typically, 5 g of nitrate powder was added into a crucible and transferred to the muffle furnace at a temperature of 350 °C (for sodium nitrate, NaNO_3_) or 380 °C (for potassium nitrate, KNO_3_) for ∼10 min. As the nitrate became molten, 0.2 g of manganese sulfate (MnSO_4_) powder was added into the molten salt for 1 min. Then, the product was removed from the muffle furnace and cooled to room temperature under ambient conditions. Finally, the product was washed with DI water to remove NaNO_3_ or KNO_3_.

### Synthesis of 2D Li_2_WO_4_ and Na_2_W_4_O_13_

The 2D Li_2_WO_4_ and Na_2_W_4_O_13_ were synthesized by the MSM. Typically, 5 g of nitrate powder was added into a crucible and transferred to the muffle furnace at a temperature of 420 °C (for lithium nitrate, LiNO_3_) or 350 °C (for NaNO_3_) for approximately 10 min. As the nitrate became molten, 0.2 g of ammonium tungstate hydrate (H_40_N_10_O_41_W_12_·*x*H_2_O) powder was added into the molten salt for 1 min. Then, the product was removed from the muffle furnace and cooled to room temperature. Finally, the product was washed with DI water to remove LiNO_3_ or NaNO_3_.

### Synthesis of 2D Zn_5_(OH)_8_(NO_3_)_2_·2H_2_O

The 2D Zn_5_(OH)_8_(NO_3_)_2_·2H_2_O was synthesized by the MSM. Typically, 5 g of KNO_3_ was added into a crucible and transferred to the muffle furnace at a temperature of 380 °C for ∼10 min. As the KNO_3_ became molten, 0.2 g zinc chloride (ZnCl_2_) powder was added into the molten salt for 1 min. Then, the product was removed from the muffle furnace and cooled to room temperature. Finally, the product was washed by DI water to remove KNO_3_.

### Synthesis of 2D Cu_2_(OH)_3_NO_3_

The 2D Cu_2_(OH)_3_NO_3_ was synthesized by MSM. Typically, 5 g KNO_3_ was added into the crucible and transferred to the muffle furnace with a temperature of 380 °C for ∼10 min. As the KNO_3_ became the molten state, 0.2 g copper nitrate trihydrate powder (Cu(NO_3_)_2_·3H_2_O) was added into the molten salt for 1 min. Then, the product was removed from muffle furnace and cooled to room temperature. Finally, the product was washed with DI water to remove KNO_3_.

### Fabrication of aqueous and solid-state devices for electrochemical tests

We used a typical three-electrode configuration in Swagelok cells (Swagelok, USA) for aqueous electrolyte testing. In this setup, YP-50 (Kuraray, Japan) was the counter electrode, Ag/AgCl (CHI, USA) was the reference electrode, 0.5 mol l^−1^ Na_2_SO_4_ solution was the electrolyte and a Celgard film served as the separator (Celgard, USA). The electrode was prepared by vacuum filtering the Na_2_W_4_O_13_ suspension (1 mg ml^−1^) on a Celgard film with a loading mass of 0.31 mg cm^−2^. The thickness of the Na_2_W_4_O_13_ film was 1 μm. The CNT ink was prepared by probe-sonicating 50 ml of a CNT (5 mg l^−1^) and sodium dodecylbenzenesulfonate (0.1 mg l^−1^) dispersion for 20 min. Na_2_W_4_O_13_ suspension (1 mg l^−1^) was prepared using a similar probe-sonication method. After coating the CNT ink and Na_2_W_4_O_13_ suspension on A4 paper separately, the SC electrode was prepared successfully. H_3_PO_4_/PVA gel electrolyte was fabricated by mixing 6 g of H_3_PO_4_ and 6 g of PVA powder with 60 ml DI water. The mixture was heated to 85 °C under vigorous stirring. The solid-state device was fabricated in two steps. First, two A4 paper@CNT@Na_2_W_4_O_13_ electrodes were dipped into H_3_PO_4_/PVA gel electrolyte for 5 min and sandwiched with a Celgard film as separator. Second, after drying at room temperature for 24 h, the solid-state device was successfully assembled. The mass loading of Na_2_W_4_O_13_ on the A4 paper@CNT was 1.2 mg cm^−2^. The thickness of the Na_2_W_4_O_13_ film was 6 μm. All of the electrochemical tests were carried out using EC-Lab and CHI660E, and electrochemical impedance was measured with a potential amplitude of 10 mV from 1 to 1 MHz.

### Characterization

X-ray diffraction (XRD, X’Pert Pro, PANalytical) was used to determine the crystal structure and stoichiometric ratio of the 2D materials, and X-ray photoelectron spectroscopy (XPS, ESCALab 250), field-emission scanning electron microscopy (FE-SEM, FEI Nova 450 Nano), transmission electron microscopy with energy dispersive X-ray spectroscopy (Tecnai G2 20 U-Twin and Titan G2 60-300), and atomic force microscopy (AFM, Shimadzu) were used to explore the structure and morphology of the samples. Zeta potential was measured using a Zetasizer (Nano ZSP, Malvern Instruments Limited, UK). N_2_ adsorption-desorption isotherms were measured on a Micrometrics ASAP 2000 to confirm the specific surface area. An SL200B contact angle metre (Kino Industry, USA) was employed to measure the surface wettability with electrolyte. The mass loading of the electrode was measured by a microbalance (CPA225D, Sartorius).

### Data availability

The authors declare that the data supporting the findings of this study are available within the paper.

## Additional information

**How to cite this article:** Hu, Z. *et al*. Rapid mass production of two-dimensional metal oxides and hydroxides via the molten salts method. *Nat. Commun.* **8**, 15630 doi: 10.1038/ncomms15630 (2017).

**Publisher’s note**: Springer Nature remains neutral with regard to jurisdictional claims in published maps and institutional affiliations.

## Supplementary Material

Supplementary Information Supplementary Figures, Supplementary Tables, Supplementary Notes and Supplementary References.

Supplementary Movie 1 Process for synthesis of two-dimensional metal oxides and hydroxides via the molten salts method
