# Supplementary material for: Rapid mass production of two-dimensional metal oxides and hydroxides via the molten salts method
Source: Nat Commun. 2017 May 30;8:15630. doi: 10.1038/ncomms15630 (PMC5499201; doi:10.1038/ncomms15630)
Supplement: Supplementary Information — Supplementary Figures, Supplementary Tables, Supplementary Notes and Supplementary References. [file ncomms15630-s1.pdf]

Supplementary Information

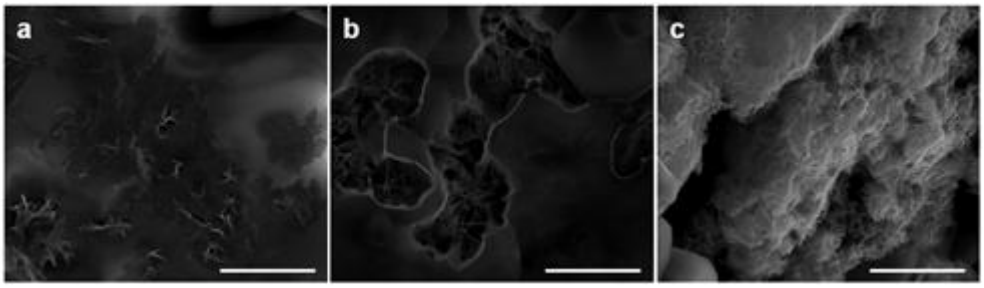

Supplementary Figure 1 │ SEM images of the final mixture after reaction without washing with deionized water (DI) water. The mixture contains the two-dimensional (2D) material ($Na_{0.55}Mn_2O_4 \cdot 1.5H_2O$), recrystallized salt ($NaNO_3$) and the byproduct ($Na_2SO_4$). Scale bar, 3 µm.

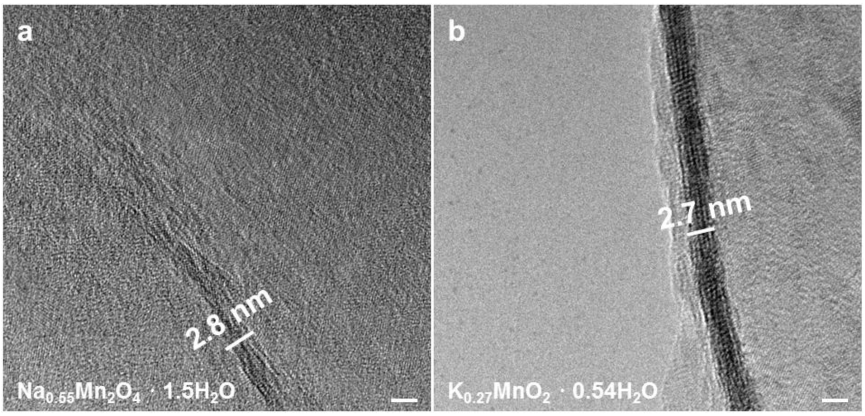

Supplementary Figure 2│Thickness measurement of 2D ion-intercalated metal oxide. For $Na_{0.55}Mn_2O_4 \cdot 1.5H_2O$ and $K_{0.27}MnO_2 \cdot 0.54H_2O$, they were both very flexible. We tested the thickness of wrinkle which was twice of the actual thickness from high resolution transmission electron microscopy (HRTEM) images. a, $Na_{0.55}Mn_2O_4 \cdot 1.5H_2O$. b, $K_{0.27}MnO_2 \cdot 0.54H_2O$. Scale bar, 2 nm.

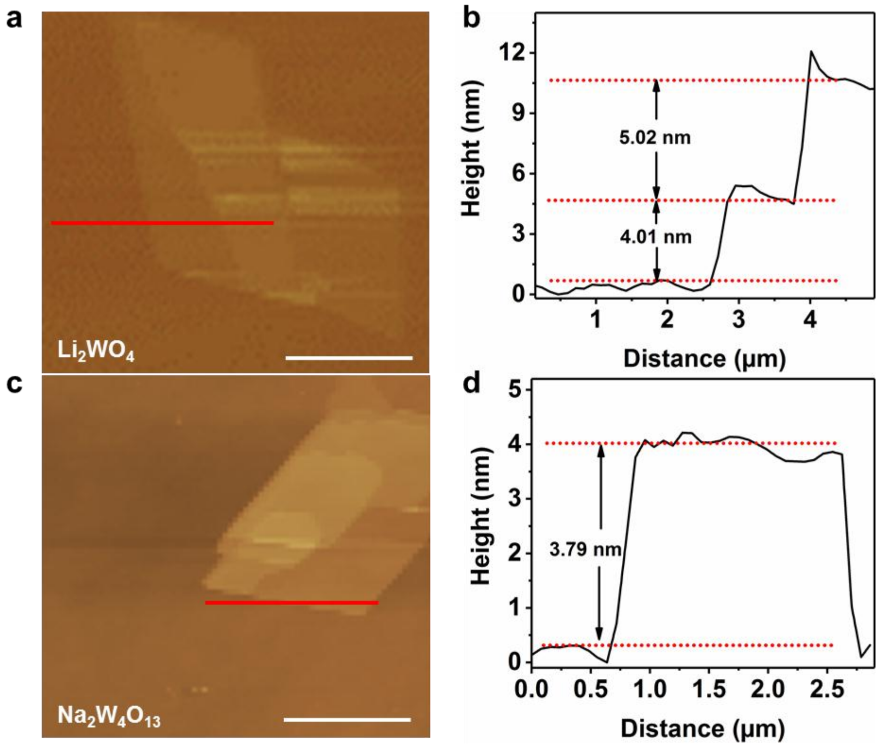

Supplementary Figure 3 | Thickness measurement of 2D ion-intercalated metal oxide. For Li_2WO_4 and $Na_2W_4O_{13}$, they were both stiff which can be tested by atomic force microscope (AFM). a, AFM image of Li_2WO_4. b, thickness of Li_2WO_4. c, AFM image of $Na_2W_4O_{13}$. d, thickness of $Na_2W_4O_{13}$. Scale bar in a and c, 2 μm.

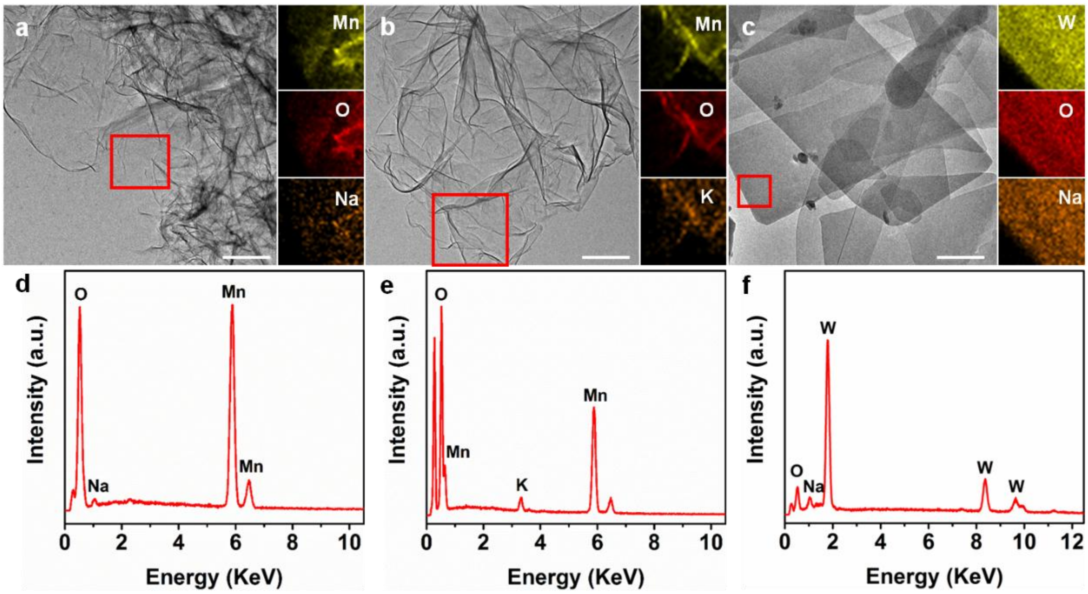

Supplementary Figure 4│Characterization of 2D ion-intercalated metal oxide. a-c, TEM images and element mapping of $Na_{0.55}Mn_2O_4 \cdot 1.5H_2O$, $K_{0.27}MnO_2 \cdot 0.54H_2O$ and $Na_2W_4O_{13}$. d-f, Energy disperse spectroscopy (EDS) spectrums of $Na_{0.55}Mn_2O_4 \cdot 1.5H_2O$, $K_{0.27}MnO_2 \cdot 0.54H_2O$ and $Na_2W_4O_{13}$. Scale bar in a - c, 200 nm.

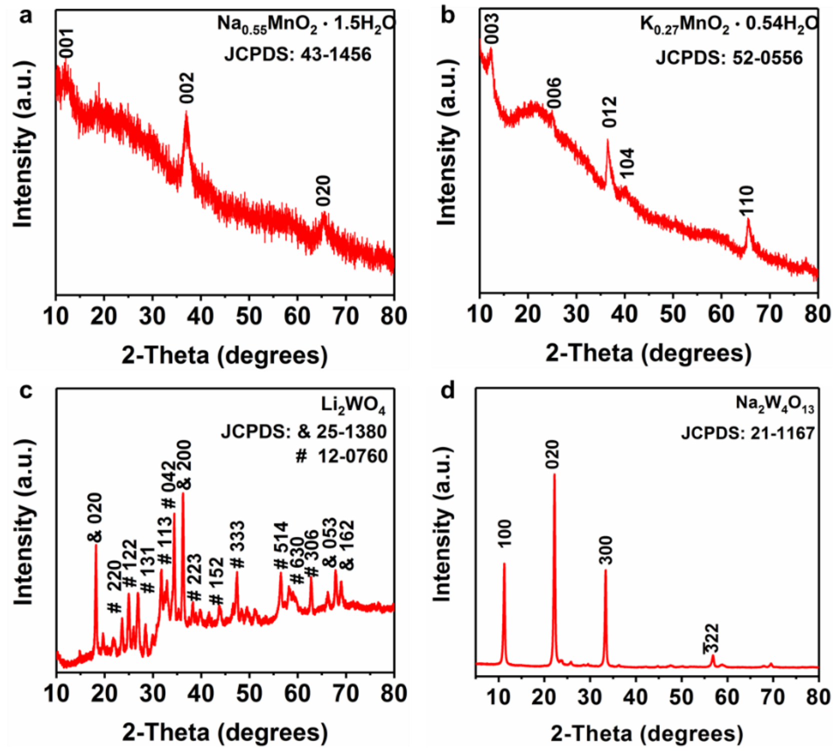

Supplementary Figure 5│X-ray diffraction (XRD) patterns of 2D ion-intercalated metal oxide. a, $Na_{0.55}Mn_2O_4$ ·$1.5H_2O$, the corresponding JCPDS card number is 43-1456. b, $K_{0.27}MnO_2$ ·$0.54H_2O$, the corresponding JCPDS card number is 52-0556. c, Li_2WO_4, the corresponding JCPDS card number is 25-1380 and 12-0760. d, $Na_2W_4O_{13}$, the corresponding JCPDS card number is 21-1167.

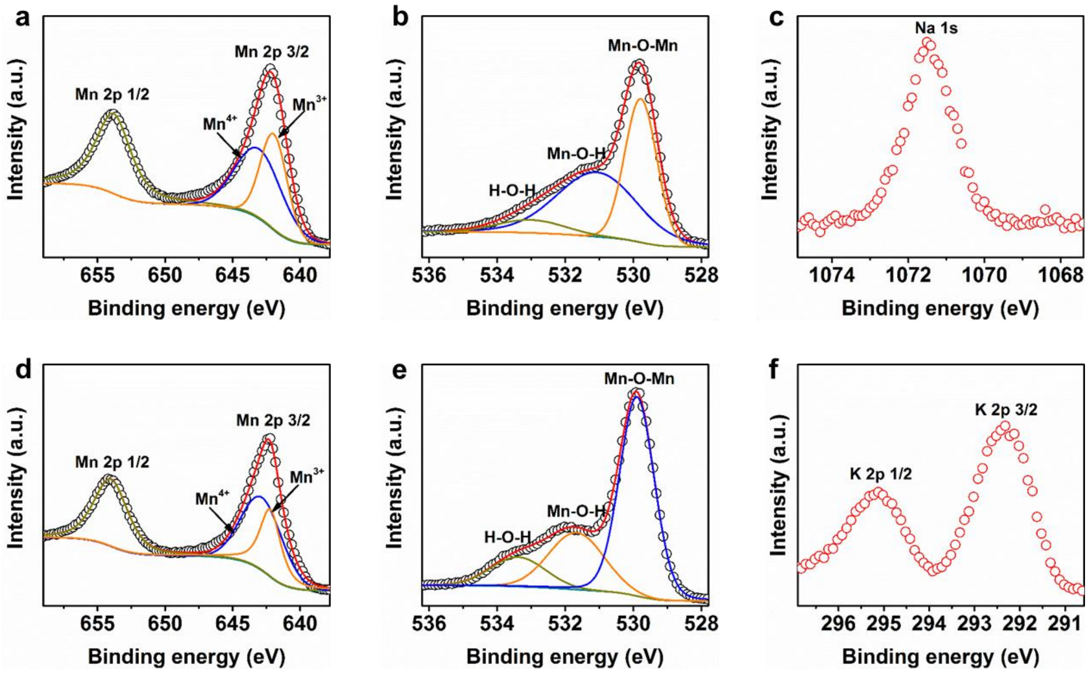

Supplementary Figure 6│XPS spectrums of 2D ion-intercalated metal oxide. a, Mn 2p; b, O1s; c, Na 1s spectrum of 2D $Na_{0.55}Mn_2O_4 \cdot 1.5H_2O$. d, Mn 2p; e, O1s; f, K 1s spectrum of 2D $K_{0.27}MnO_2 \cdot 0.54H_2O$. The Mn 2p 3/2 can split to two peaks due to the surface defects, corresponding to Mn^{3+}-O and Mn^{4+}-O while the O 1s can split into three peaks, corresponding to Mn-O-Mn, Mn-O-H and H-O-H.

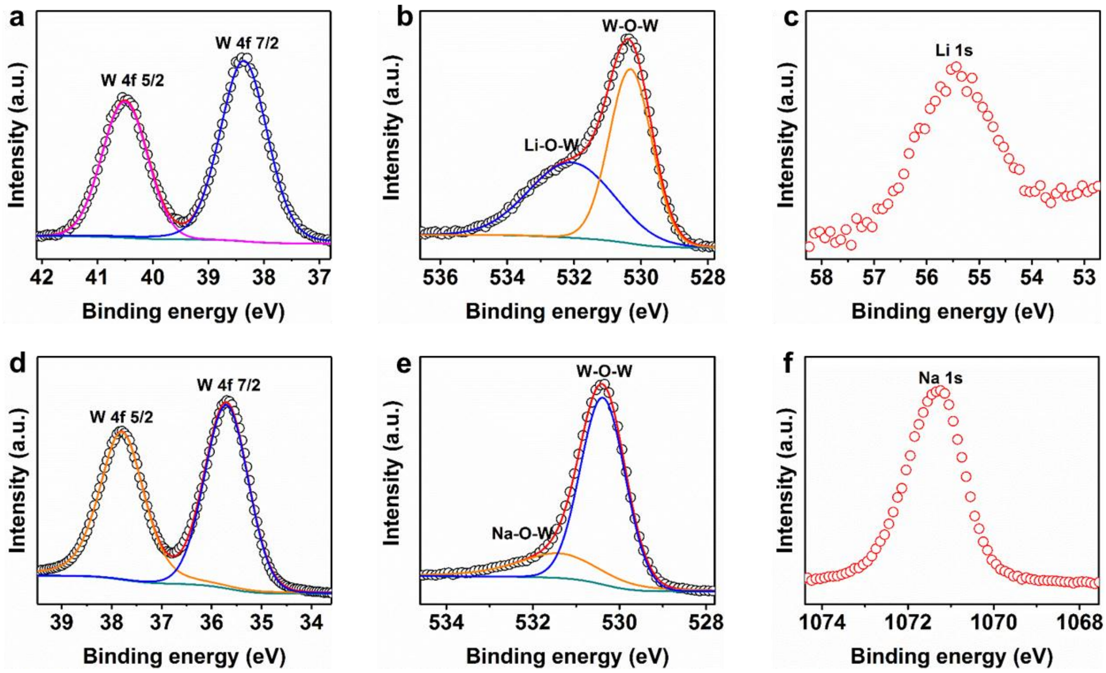

Supplementary Figure 7 | XPS spectrums of 2D ion-intercalated metal oxide. a, W 4f; b, O1s; c, Li 1s spectrum of 2D Li_2WO_4. d, W 4f; e, O1s; f, Na 1s spectrum of 2D $Na_2W_4O_{13}$. The O 1s can split into two peaks, corresponding to W-O-W and Na-O-W.

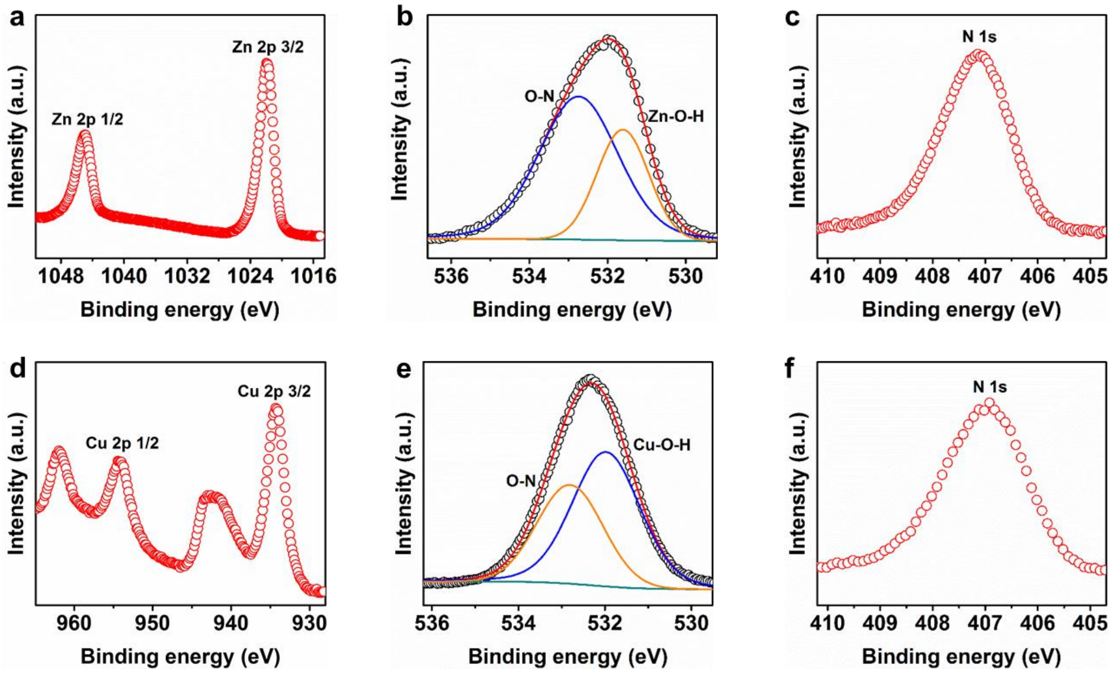

Supplementary Figure 8 | XPS spectrums of 2D ion-intercalated metal hydroxide. a, Zn 2p; b, O1s; c, N 1s spectrum of 2D $Zn_5(OH)_8(NO_3)_2 \cdot 2H_2O$. d, Cu 2p; e, O1s; f, N 1s spectrum of 2D $Cu_2(OH)_3NO_3$. The O 1s can split into two peaks, corresponding to O-N and Zn-O-H for sample $Zn_5(OH)_8(NO_3)_2 \cdot 2H_2O$ and corresponding to O-N and Cu-O-H for sample $Cu_2(OH)_3NO_3$.

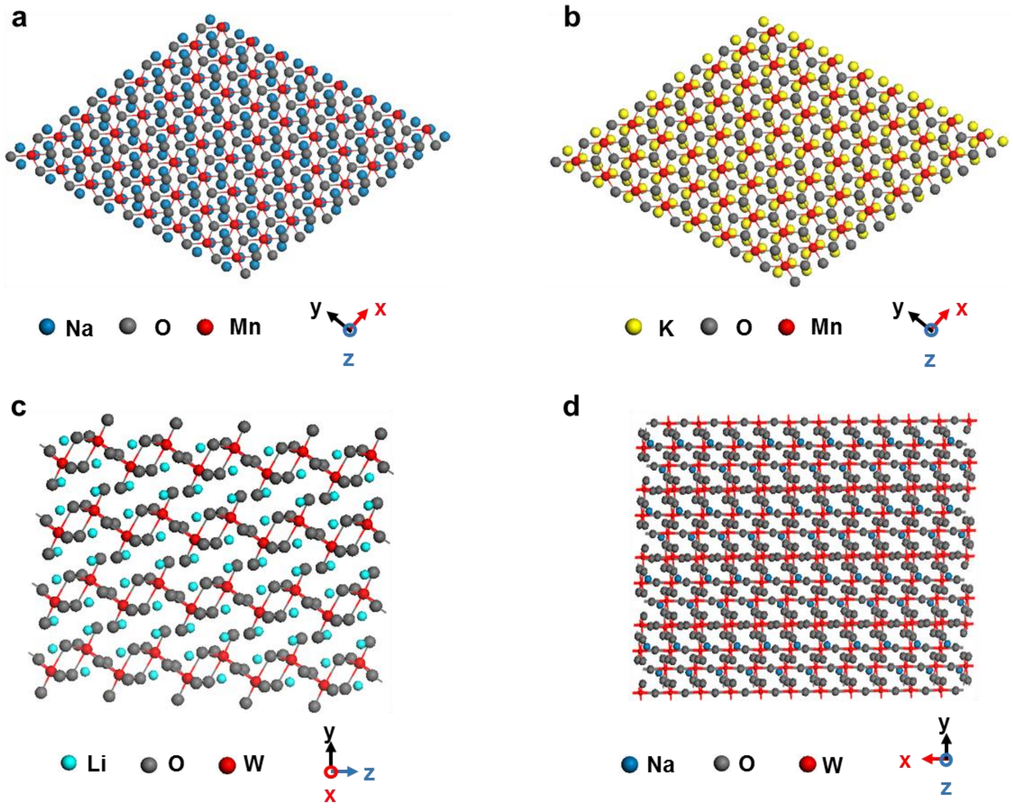

Supplementary Figure 9│Top view atomic structures of 2D cation-intercalated metal oxide. a, $Na_{0.55}Mn_2O_4 \cdot 1.5H_2O$. b, $K_{0.27}MnO_2 \cdot 0.54H_2O$. c, Li_2WO_4. d, $Na_2W_4O_{13}$.

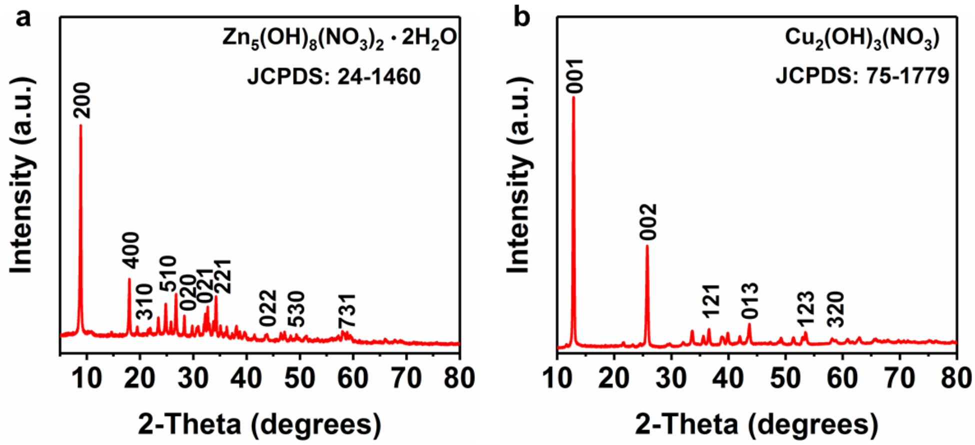

Supplementary Figure 10 | XRD patterns of 2D ion-intercalated metal hydroxide. a, $Zn_5(OH)_8(NO_3)_2 \cdot 2H_2O$, the corresponding JCPDS card number is 24-1460. b, $Cu_2(OH)_3NO_3$, the corresponding JCPDS card number is 75-1779.

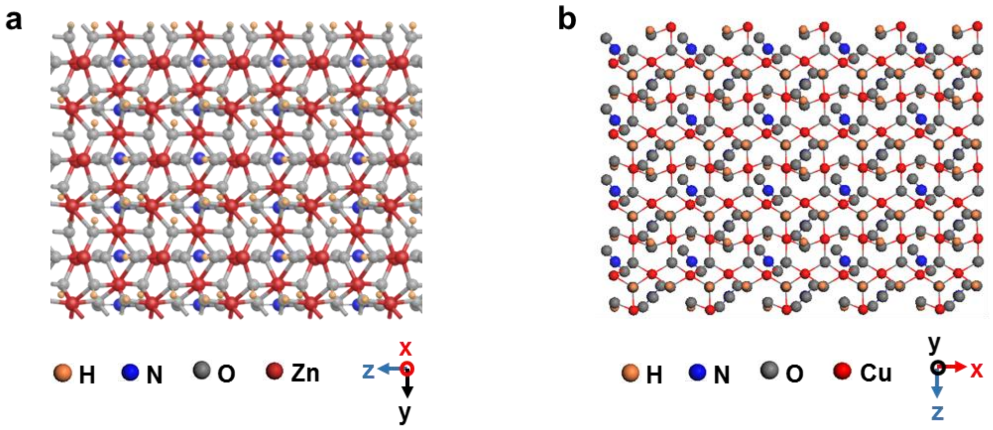

Supplementary Figure 11│Top view atomic structures of 2D anion-intercalated metal hydroxide. a, $Zn_5(OH)_8(NO_3)_2\cdot 2H_2O$. b, $Cu_2(OH)_3NO_3$.

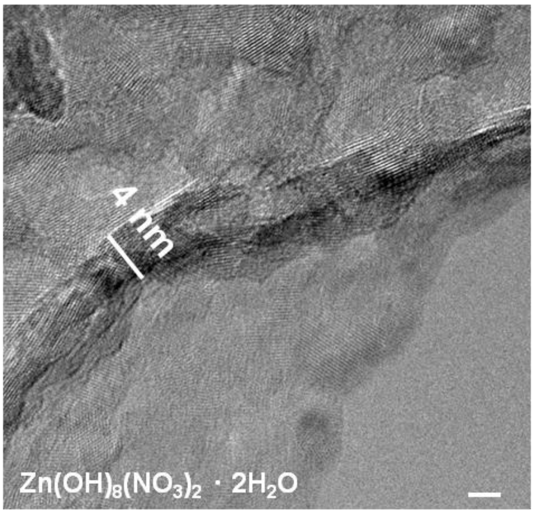

Supplementary Figure 12 │ Thickness measurement of 2D $Zn_5(OH)_8(NO_3)_2 \cdot 2H_2O$. The thickness of the wrinkle is 4 nm which means the thickness of $Zn_5(OH)_8(NO_3)_2 \cdot 2H_2O$ is 2 nm from HRTEM image. Scale bar, 2 nm.

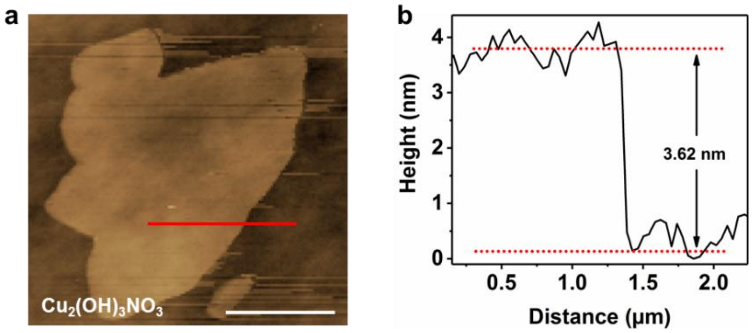

Supplementary Figure 13 | Thickness measurement of 2D $Cu_2(OH)_3NO_3$ by AFM. a, AFM image of $Cu_2(OH)_3NO_3$. b, thickness of $Cu_2(OH)_3NO_3$. Scale bar in a, 2 μm.

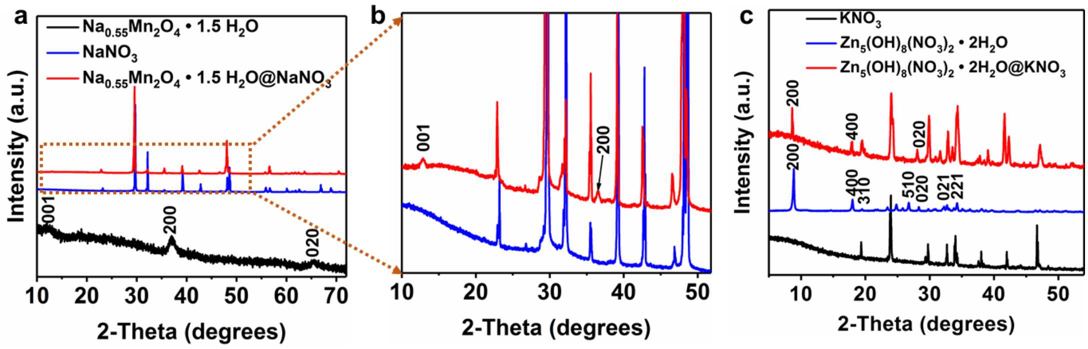

Supplementary Figure 14 | XRD patterns of 2D ion-intercalated materials before and after washing with DI water. a, $Na_{0.55}Mn_2O_4 \cdot 1.5H_2O$. b, $Zn_5(OH)_8(NO_3)_2 \cdot 2H_2O$. According to XRD patterns, we believed the crystal water in these 2D metal oxides and hydroxides was formed during the growth process rather than the washing process, because the crystal structure didn't show any change before and after washing out the salts.

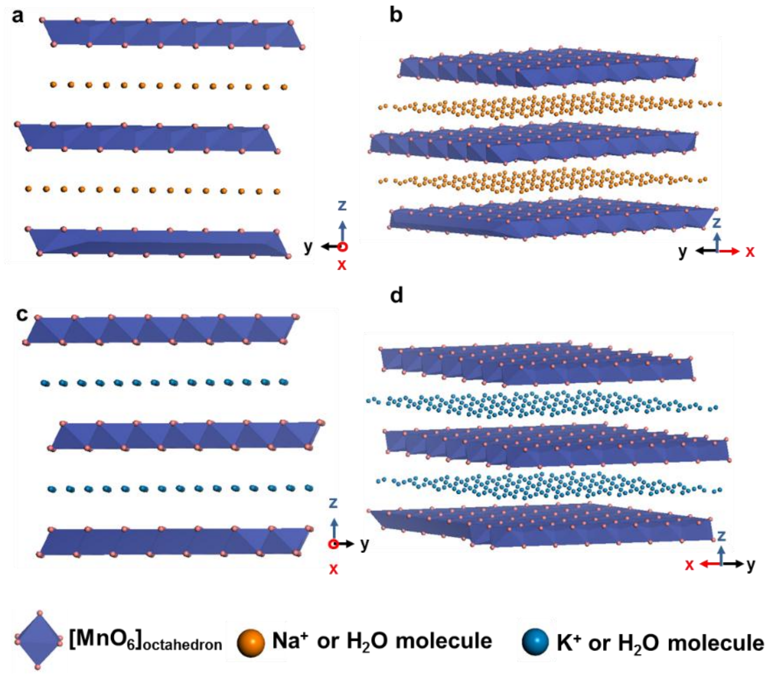

Supplementary Figure 15│Crystal structures of $Na_{0.55}Mn_2O_4 \cdot 1.5H_2O$ and $K_{0.27}MnO_2$ $0.54H_2O$. a and b, The crystal structure of $Na_{0.55}Mn_2O_4 \cdot 1.5H_2O$ with different viewing angle. c and d, The crystal structure of $K_{0.27}MnO_2$ $0.54H_2O$ with different viewing angle.

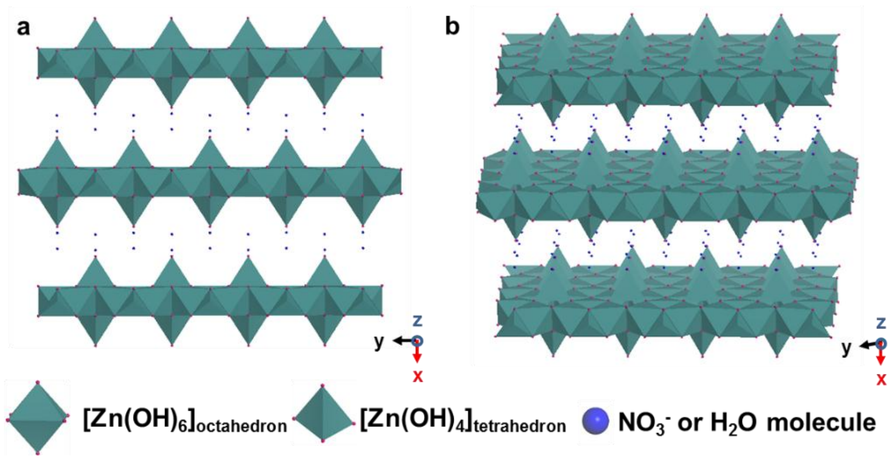

Supplementary Figure 16 | Crystal structure of $Zn_5(OH)_8(NO_3)_2 \cdot 2H_2O$. a and b show the crystal structure of $Zn_5(OH)_8(NO_3)_2 \cdot 2H_2O$ with different viewing angle.

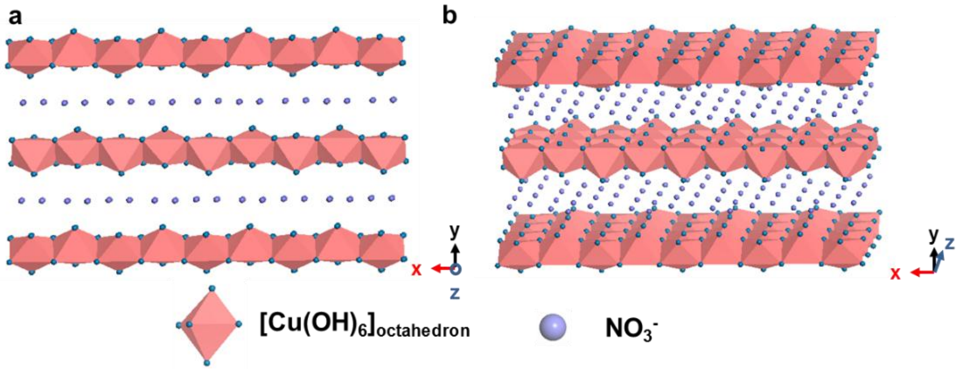

Supplementary Figure 17 | Crystal structures of $Cu_2(OH)_3NO_3$. a and b show the crystal structure of $Cu_2(OH)_3NO_3$ with different viewing angle.

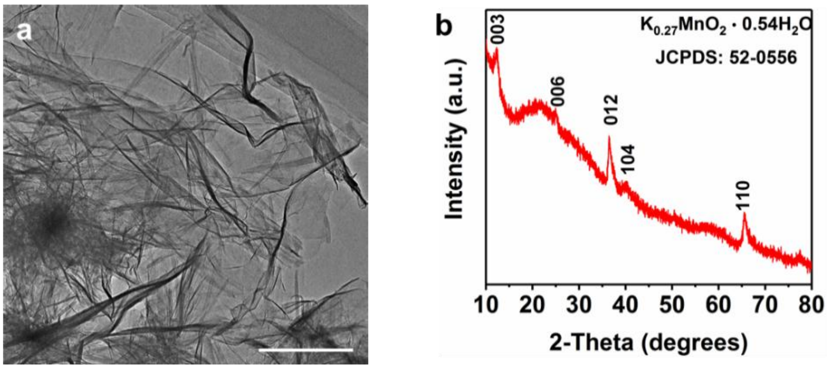

Supplementary Figure 18 | Morphology and structure information of manganese oxides. a, TEM image of δ-MnO_2 as the reaction time prolonging to 10 minutes. b, XRD pattern of δ-MnO_2 as the reaction time prolonging to 10 minutes, the corresponding JCPDS card number is 52-0556. Scale bar, 200 nm.

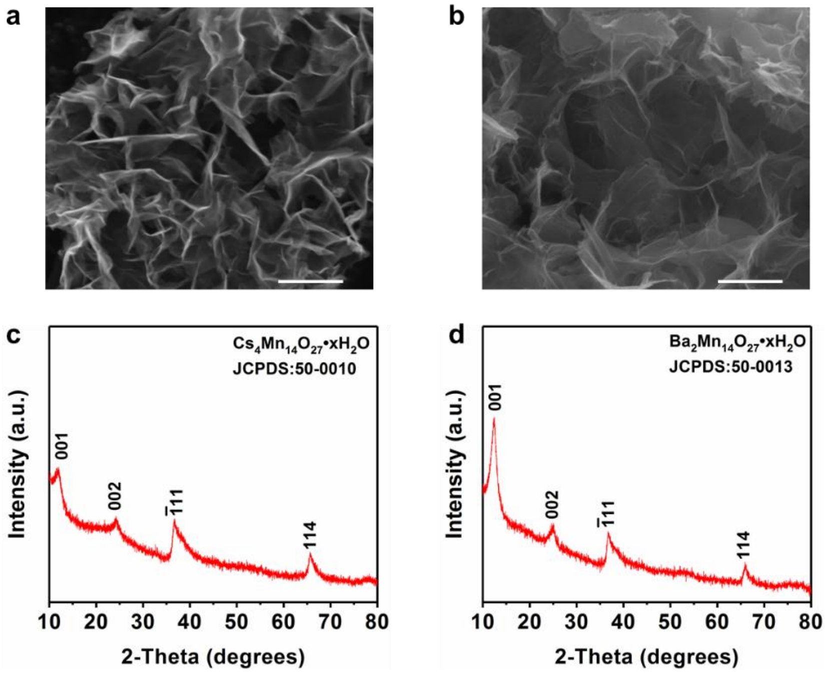

Supplementary Figure 19│Morphology and structure information of $Cs_4Mn_{14}O_{27}\cdot xH_2O$ and $Ba_2Mn_{14}O_{27}\cdot xH_2O$. a, SEM images of $Cs_4Mn_{14}O_{27}\cdot xH_2O$. b, SEM image of $Ba_2Mn_{14}O_{27}\cdot xH_2O$. c, XRD pattern of $Cs_4Mn_{14}O_{27}\cdot xH_2O$. d, XRD pattern of $Ba_2Mn_{14}O_{27}\cdot xH_2O$. In a typical experiment, 0.5g $CsNO_3$ and $Ba(NO_3)_2$ was added into the crucible and transferred to the muffle furnace with a temperature of 450 ℃ (for $CsNO_3$) or 620 ℃ ($Ba(NO_3)_2$) for about 10 minutes. As the nitrate became the molten solution, 0.2 g $MnSO_4$ power was added into the molten salt for 1 minute. Then, the product was moved out from muffle furnace and cooled to room temperature under ambient condition. Finally, the product was washed by DI water to remove $CsNO_3$ or $Ba(NO_3)_2$ and byproduct. Scale bar, 1 μm

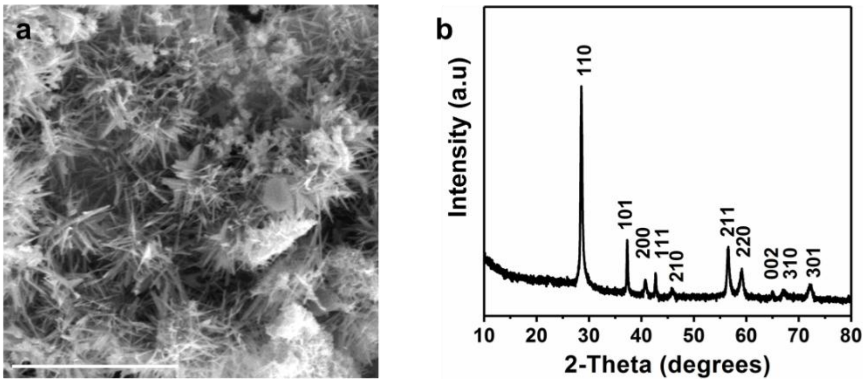

Supplementary Figure 20 | Morphology and structure of MnO_2 with the $LiNO_3$ as molten salt. When we used $LiNO_3$ as the molten salt, we obtain nanowires instead of 2D Li-intercalated MnO_2. a, SEM image of MnO_2. b, XRD pattern of MnO_2, the corresponding JCPDS card number is 81-2261 and the crystal structure belongs to β-MnO_2. Scale bar, 2 μm.

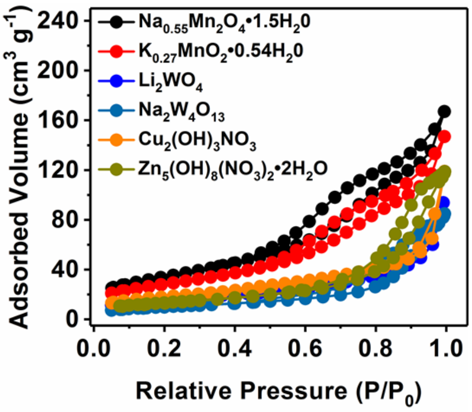

Supplementary Figure 21 | N_2 adsorption and desorption isotherms of 2D ion-intercalated materials. Including $Na_{0.55}Mn_2O_4 \cdot 1.5H_2O$, $K_{0.27}MnO_2\ 0.54H_2O$, Li_2WO_4, $Na_2W_4O_{13}$, $Cu_2(OH)_3NO_3$ and $Zn_5(OH)_8(NO_3)_2 \cdot 2H_2O$.

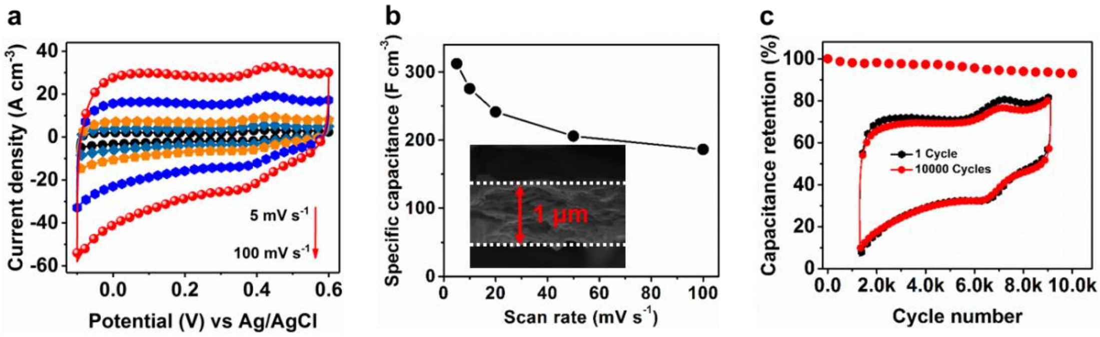

Supplementary Figure 22 | Electrochemical performance of 2D $Na_2W_4O_{13}$ electrode in 0.5 mol L^{-1} Na_2SO_4 electrolyte. a, CV curves at different sweep rate from 5 mV s^{-1} to 100 mV s^{-1}. b, Volumetric capacitance versus different sweep rate. The inset is the cross-sectional SEM image of the electrode. c, Long-term stability of the electrode. The inset is the CV curves of the first cycle and the 10000th cycle.

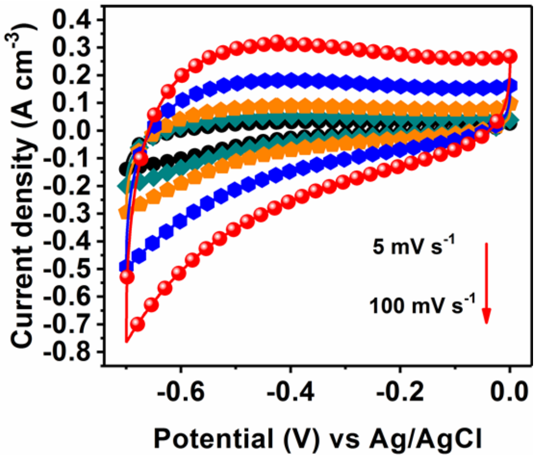

Supplementary Figure 23 | Electrochemical performance of 2D $Na_2W_4O_{13}$ in 0.5 mol L^{-1} Na_2SO_4 electrolyte as negative electrode. The sweep rate from 5 mV s^{-1} to 100 mV s^{-1} and the potential window from -0.7 V to 0 V.

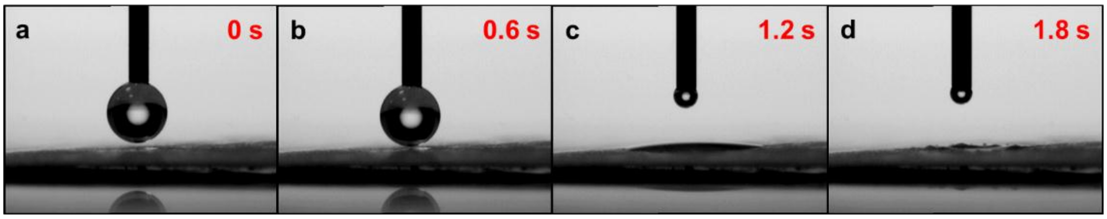

Supplementary Figure 24 | The change of contact angle with different time. a-d The contact angle of $Na_2W_4O_{13}$ film with 0.5 mol L^{-1} Na_2SO_4 solution. a, The contact time of $Na_2W_4O_{13}$ film and Na_2SO_4 solution is 0 s. b, The contact time of $Na_2W_4O_{13}$ film and Na_2SO_4 solution is 0.6 s. c, The contact time of $Na_2W_4O_{13}$ film and Na_2SO_4 solution is 1.2 s. d, The contact time of $Na_2W_4O_{13}$ film and Na_2SO_4 solution is 1.8 s.

a b

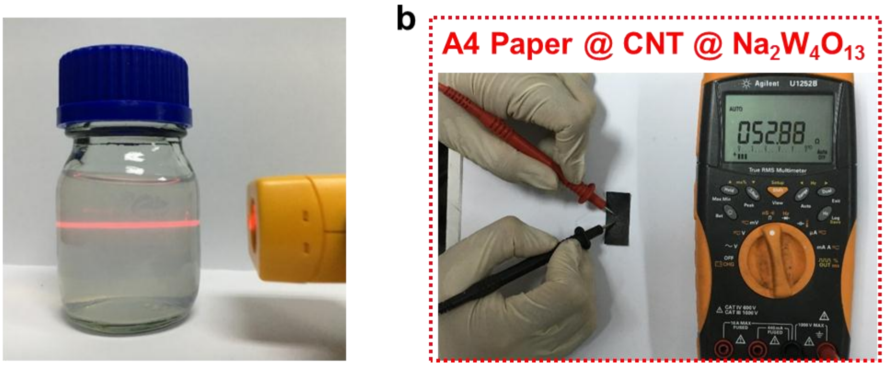

Supplementary Figure 25│Tyndall effect of the $Na_2W_4O_{13}$ dispersion and electron conductivity of the electrodes. a, Obvious Tyndall effect was observed for $Na_2W_4O_{13}$ dispersion. b, Electron conductivity of the A4 paper@CNT@$Na_2W_4O_{13}$ electrodes.

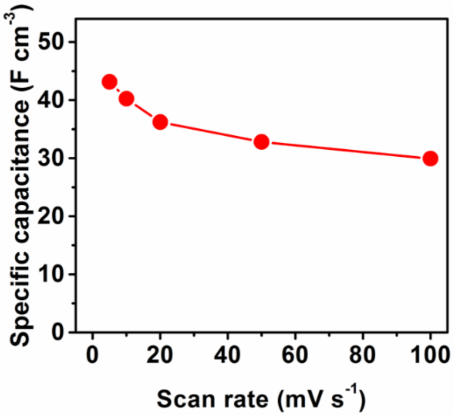

Supplementary Figure 26 | Capacitance of the solid-state SC at different sweep rates. Volumetric capacitance value of the solid-state SC at sweep rate from 5 mV s^{-1} to 100 mV s^{-1}.

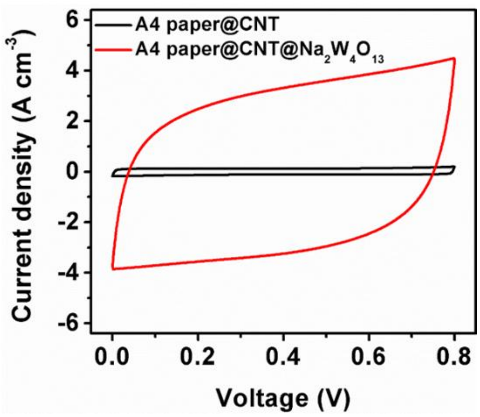

Supplementary Figure 27 | CV curves of the solid-state supercapacitor. A4 paper @ CNT or A4 paper @ CNT @ $Na_2W_4O_{13}$ as electrode materials at sweep rates of 100 mV s^{-1}.

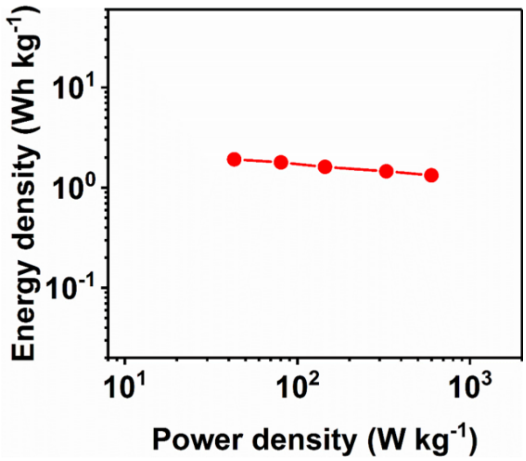

Supplementary Figure 28 | Ragone plot of fabricated solid-state supercapacitor device. The electrode for solid-stated device was prepared by coating $Na_2W_4O_{13}$ dispersion on A4 paper@CNT substrate. The mass loading of $Na_2W_4O_{13}$ on A4 paper@CNT is 1.2 mg cm^{-2}. The thickness of the $Na_2W_4O_{13}$ film is 6 μm.

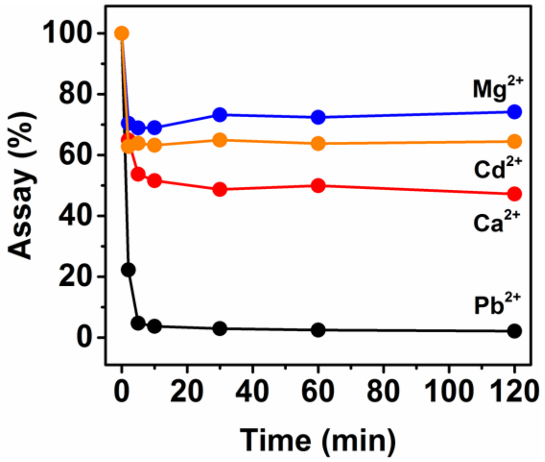

Supplementary Figure 29 | Ions adsorption of 2D $Na_2W_4O_{18}$ for various cations. In a typical adsorption experiment, 30 mg $Na_2W_4O_{13}$ powder was introduced into 100 mL bottle with 50 mL solution containing 50 mg L^{-1} metal ions (Pb^{2+}, Ca^{2+}, Cd^{2+} and Mg^{2+}) and stirring for 2 hours. Finally, the resulting solutions were filtered through 0.45 micrometers membranes.

Supplementary Table 1 | BET surface area of different 2D materials

**Specimen**	**BET surface area (m^2 g^{-1})**
$Na_{0.55}$ $Mn_2O_4 \cdot 1.5H_2O$	123.5
$K_{0.27}MnO_2$ 0.54 H_2O	102.0
Li_2WO_4	57.8
$Na_2W_4O_{13}$	35.7
$Cu_2(OH)_3NO_3$	47.4
$Zn_5(OH)_8(NO_3)_2 \cdot 2H_2O$	63.9

Supplementary Note 1. Calculation process of productivity of sample $Na_{0.55}Mn_2O_4 \cdot 1.5H_2O$.

The calculation formula of production yield is shown as following:

$$\text{Production yield} = \frac{\text{the actual weight}}{\text{the theoretical weight}} \quad (1)$$

The actual weight of $Na_{0.55}Mn_2O_4 \cdot 1.5H_2O$ was measured by a microbalance with an accuracy of 0.1 mg. The theoretical weight of $Na_{0.55}Mn_2O_4 \cdot 1.5H_2O$ was calculated based on the weight of $MnSO_4$ precursor. In this work, we used 0.2 g $MnSO_4$ as precursor, thus the theoretical weight of $Na_{0.55}Mn_2O_4 \cdot 1.5H_2O$ should be 0.1415 g, and the actual weight is 0.0877 g. The production yield of $Na_{0.55}Mn_2O_4 \cdot 1.5H_2O$ is about 62 %.

Supplementary Note 2. The source of crystal water.

According to the structural model of birnessite type manganese oxide calculated by Post and Veblen, Mn(IV) and Mn(III) atoms distributed uniformly in the manganese oxide sheet. Hence, the charge density of manganese oxide sheet can be calculated on the basis of this model[1]. In order to balance the charge density, the cations should occupy the interlayer. Besides, a certain amount of water molecules is needed to support the interlayer spacing and then stabilize the layered structure. Since the actual content of water molecules will increase as the temperature rising, it means that when the reaction temperature exceeds 300 ℃, the absolute humidity in the muffle furnace is increasing compared to starting temperature (25 ℃). So the water molecules can enter into the interlayer with cations to support the layered structure even at 300 ℃.

To discuss the source of the crystal water, we have measured the XRD to illustrate the change of the crystal structure before and after the washing step (Supplementary Fig. 14). Without washing by deionized water (DI water), the XRD pattern of the sample $Na_{0.55}Mn_2O_4 \cdot 1.5H_2O$@ $NaNO_3$ already shows the phase of $Na_{0.55}Mn_2O_4 \cdot 1.5H_2O$ and $NaNO_3$ which means the crystal water came from the reaction process but not the washing step. Similar results can be also concluded from the sample of $Zn_5(OH)_8(NO_3)_2 \cdot 2H_2O$ @ KNO_3, as shown in Supplementary Fig. 14b.

Supplementary Note 3. Arrhenius equation.

The relationship between reaction temperature (T) and reaction rate constant (k_s) can be expressed as below from the Arrhenius equation:

$$k_s = Ae^{-\frac{E_s}{RT}} \qquad (2)$$

where k_s is the reaction rate constant (s^{-1}), A is the frequency factor (s^{-1}), E_s is the activation energy (J mol^{-1}), R is the molar gas constant (8.314 J $mol^{-1}K^{-1}$) and T is the reaction temperature. To a typical 2D structure material, k_s of different growth direction decide the thickness and lateral size. k_a and k_b represent the reaction rate constant of growth dire ction [001] and [100], respectively; The thickness and lateral size depend on k_a and k_b; A is the same constant for different lattice direction and reaction systems.

The activation energy E_s should be unchanged in different reaction systems under the same temperature.

According to equation (2), we can obtain the relationship of k_b and k_a:

$$\frac{k_b}{k_a} = e^{\frac{E_a - E_b}{RT}} \qquad (3)$$

Supplementary Note 4. The influence of reaction temperature.

In Sun's work (ref 2), they have synthesized ultrathin 2D δ-MnO_2 (birnessite) nanosheets *via* a hydrothermal method. The thickness is around 2 - 10 nm and the plane size is about 1 - 2 μm, the temperature is 150 ºC (423 K).

The ratio of k_b and k_a and the difference value between E_a and E_b through the values of thickness and lateral size are calculated *via* equation (3).

$$\frac{k_b}{k_a} = 100 \text{ - } 1000 \qquad (4)$$

$$E_a - E_b = 16.2 \text{ - } 24.3 \text{ kJ mol}^{-1} \qquad (5)$$

We supposed that $E_a - E_b$ remain unchanged as the temperature raised to 653 K (the same temperature in our method), hence the ratio of k_b and k_a was calculated as 19.8 - 87.7. If the thickness was maintained at 2 nm, the lateral size of birnessite should be 39.6 - 175.5 nm. However, in our work under the same temperature, the thickness is about 1.4 nm and the lateral size is more than 10 μm, the ratio of k_b and

k_a is 7142.8 which is much larger than 87.7:

$$\frac{k_{b\prime}}{k_{a\prime}} = e^{\frac{E_{a\prime}-E_{b\prime}}{RT}} = \frac{10\ \mu\text{m}}{1.4\ \text{nm}} = 7142.8 \qquad (6)$$

Thus the lateral size cannot be enlarged through improving the reaction temperature.

Supplementary Note 5. The influence of the ion state.

Then we would discuss the influence of hydrated ion and naked ion on the lateral size of birnessite. From equation 6,

$$E_{a\prime} - E_{b\prime} = 48.2\ \text{kJ mol}^{-1} \qquad (7)$$

$E_{a\prime}$ can be considered the same as E_a due to the similar thickness and the same temperature in the two reaction systems.

$$E_b - E_{b\prime} = (E_{a'} - E_{b'}) - (E_a - E_b) = 23.9 - 32.0\ \text{kJ mol}^{-1} \qquad (8)$$

The difference value of $E_{b\prime}$ and E_b represents the extra energy along the [100] direction in hydrothermal method compared to molten salt system. As the total hydration energy of K^+ is -312.2 kJ mol^{-1}, in our work, the K^+ number is 0.27, thus the dehydration energy is about 84.3 kJ mol^{-1} to the sample of $K_{0.27}MnO_2$ $0.54H_2O$. This value has the same order of magnitude to the value of $(E_b - E_{b\prime})$ (23.9 - 32.0 kJ mol^{-1}), considering that the dehydration process cannot be incomplete in the hydrothermal system, the actual dehydration energy would be lower than 84.3 kJ mol^{-1}. Thus we can infer that the ionized ions in our method have a decisive effect on the size of the 2D materials by accelerating the reaction rate efficiently.

Supplementary Note 6. The molecular dynamics simulation of aqueous KCl solution.

The molecular dynamics (MD) simulation of aqueous KCl solution was performed in the NPT ensemble with the MD package Gromacs 4.6[3]. The SPC/E model (ref 4) was used to mimic the water molecule, which is a rigid, three-point charge model that accurately predicts the thermodynamic and structural properties of water. The K^+/Cl^- ions were modeled as charged Lennard-Jones (LJ) atoms with unit

charge and the LJ parameters for the K^+ and Cl^- were taken from ref 5. The simulation system consists of 6000 water molecules with 20 pairs of KCl ions in a cubic box (Supplementary Fig. 30), which could be seen as a dilute solution (that is the interaction between K^+ and Cl^- could be neglected). Periodic boundary conditions were used in all three directions. The temperature was maintained at 298 K and 423 K using the Nosé-Hoover thermostat with a relaxation time of 0.5 ps. And the Parrinello-Rahman barostat was used to maintain the pressure of 1 bar for 298 K and 4.76 bar (ref 6) for 423 K, respectively, with a time constant of 1 ps. The electrostatic interactions were computed using the PME method[7]. To compute the interactions in the reciprocal space, an FFT grid spacing of 0.1 nm and cubic interpolation for charge distribution were used. A cutoff distance of 1.2 nm was used in the calculation of electrostatic interactions in the real space. And the leapfrog integration algorithm was used with a time step of 2 fs to solve the equations of motion. The trajectory was saved every 2 ps. Each simulation was equilibrated within 4 ns. After that, another 2 ns production run was performed for analysis.

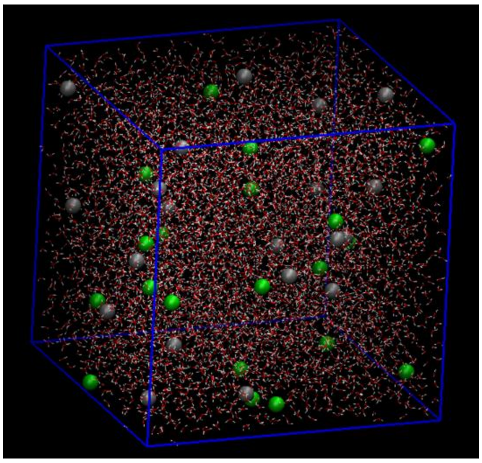

Supplementary Figure 30 | The molecular dynamics simulation of aqueous KCl solutions. The simulation system consists of 6000 water molecules with 20 pairs of KCl ions in a cubic box.

From the MD simulation of aqueous KCl solutions, the average co-number of water molecule around K^+ can be calculated in Supplementary Table 2. The total hydration energy of K^+ is -326.8 kJ mol^{-1}, -312.2 kJ mol^{-1} under 298 K and 423 K

(Supplementary Table 3) respectively.

Supplementary Table 2 | The number of water molecule around K^+ ion under different temperature

T	Co-number
298 K	8.3488
423 K	7.6948

Supplementary Table 3 | The hydration energy (E_{tot}) of K^+ with one water molecule under different temperature

T	E_{vdW}(kJ mol^{-1})	E_{coul}(kJ mol^{-1})	E_{tot}(kJ mol^{-1})
298 K	3.9095	-43.054	**-39.144**
423 K	4.1295	-44.704	**-40.574**

Supplementary Note 7. Electrode reaction for both positive and negative electrodes.

The probable electrode reaction for positive and negative electrode is shown as following:

$$Na_2W_4O_{13}+xNa^++xe^- \leftrightarrow Na_{2+x}W_4O_{13} \quad (-2 \leq x \leq 4) \quad (9)$$

The equation express the reaction of negative electrode as $-2 \leq x < 0$ while represents the reaction of positive electrode as $0 < x \leq 4$.

Supplementary Note 8. Calculation of specific energy/power density.

The specific energy and power density based on following equations (10-13).

$$E_v = 0.5C_vU^2 \quad (10)$$

$$P_v = 3600E_v/t \quad (11)$$

Where C_v (F cm^{-3}) is the volumetric capacitance of the device. E_v (Wh cm^{-3})

and P_v (W cm^{-3}) is the volumetric energy density and volumetric power density, respectively. U (V) is the operating voltage of the device and t (h) is the discharging time.

$$E_g = 0.5C_gU^2 \quad (12)$$

$$P_g = 3600E_g/t \quad (13)$$

Where C_g (F g^{-1}) is the gravimetric capacitance of the device. E_g (Wh kg^{-1}) and P_g (W kg^{-1}) is the gravimetric energy density and gravimetric power density, respectively. U (V) is the operating voltage of the device and t (h) is the discharging time. The gravimetric specific energy/power densities are shown in Supplementary Fig. 25. The gravimetric specific energy density reaches 1.33 Wh kg^{-1} while the specific power density is 598.4 W kg^{-1}.

Supplementary References

1. Liu, Z. H. *et al.* Swelling and delamination behaviors of birnessite-type manganese oxide by intercalation of tetraalkylammonium ions. *Langmuir* **16**, 4154-4164 (2000).
2. Sun, Y. G. *et al.* Birnessite-type MnO_2 nanosheets with layered structure under high pressure: Elimination of crystalline stacking faults and oriented laminar assembly. *Small* **11**, 300-305 (2015).
3. Van der Spoel, D. *et al.* GROMACS User Manual version (2013).
4. Berendsen, H. J. C. *et al.* The missing term in effective pair potentials. *J. Phys. Chem.* **91**, 6269-6271 (1987).
5. Dang, L. X., Free Energy of association of the K^+:18-crown-6 complex in water: A new molecular dynamics study. *J. Phys. Chem.* **99**, 55-58 (1995).
6. Sonntag, R. E. *et al.* Van Wyk, Fundamentals of thermodynamics. **6**, Wiley New York (1998).
7. Yeh, I. C. *et al.* Ewald summation for systems with slab geometry. *J. Chem. Phys.* **111**, 3155-3162 (1999).
8. Sun, Y. *et al.* Graphene based new energy materials. *Energy Environ. Sci.* **4**,

1113-1132 (2011).

9. Choi, H. J. *et al.* Graphene for energy conversion and storage in fuel cells and supercapacitors. *Nano Energy* **1**, 534-551 (2012).
